# A Clinician's Brief Guide to the Mental Capacity Act (2nd edn)

**DOI:** 10.1192/pb.bp.116.054783

**Published:** 2017-02

**Authors:** Martin Curtice

This book aims to provide a comprehensive overview of the Mental Capacity Act 2005 (MCA) – which applies specifically to England and Wales – and its implementation in practice.

The authors are all practising psychiatrists and although the style and content is tailored for a medical readership, the guide is suitable for all grades of doctors and all specialties, not just psychiatry. It is also ideal for medical and nursing students. With a punchy and concise writing style, the book has copious amounts of practical advice for clinicians throughout, and at times uses a common sense question-and-answer format with questions that clinicians are likely to pose, which reflects real-life practice.

Importantly, this work sought to translate lengthy and wordy court judgments into concise and simplified reviews outlining key basic principles for clinicians to use in daily practice. Possibly the most interesting chapter was that regarding the role of the Court of Protection. This busy court – which according to the authors hears approximately 23 000 cases annually, a figure that will surely inevitably rise – is often referred to in the media as the secretive court. However, this excellent chapter goes a long way in debunking various perceptions. It also explains the court process and is infused with sage, detailed and practical advice, from how to handle requests for assessments, writing reports and interviewing patients to giving evidence in court and even finding your way there if you need to! The authors suggest that Court of Protection proceedings tend to be more ‘informal and inquisitorial than formal and adversarial’ but that they can still be stressful, which is why they wish readers ‘good luck’. But despite the suggestion that a degree of luck might be needed, anyone new to such court proceedings will be far more prepared having read this chapter than not.

Needless to say, the thorny issue of implementing Deprivation of Liberty Safeguards (DoLS) was discussed at length in a chapter that provided important context by describing the evolution of this legislation and case law. Notwithstanding, owing to a glut of more recent key DoLS judgments, the book is already a little out of date as DoLS case law and guidance have evolved rapidly. It seems likely that an update will be needed soon to keep readers informed of key developments. Nevertheless, there was a good description of practical issues in using and applying DoLS since the *Cheshire West* case in 2014, a case which triggered an upsurge in the use of this legislation. The authors aptly summed up the state of DoLS understanding from further case law since *Cheshire West* by saying it did ‘little to ease the quandaries of health and social care staff in their decision-making in relation to deprivation of liberty’.

Another notable chapter was the one on the assessment of capacity, which provided comprehensive and practical advice, breaking the process down into its components and getting into its minutiae, thus challenging the reader to re-evaluate their own methods for assessing capacity. Other useful sections included advice on how to resolve conflict emanating from complex best-interests meetings and on seeking consent. Although not concluded at the time of publication, the latter resonates with the 2015 seminal Supreme Court case of *Montgomery v Lanarkshire* which has redefined the rules of seeking consent and has implications for how clinical negligence will hence be assessed.

All in all, this is an excellent guide which would aid those involved in care touching upon the use of the MCA.

